# β2-adrenoceptor signaling regulates invadopodia formation to enhance tumor cell invasion

**DOI:** 10.1186/s13058-015-0655-3

**Published:** 2015-11-25

**Authors:** Sarah J. Creed, Caroline P. Le, Mona Hassan, Cindy K. Pon, Sabine Albold, Keefe T. Chan, Matthew E. Berginski, Zhendong Huang, James E. Bear, J. Robert Lane, Michelle L. Halls, Davide Ferrari, Cameron J. Nowell, Erica K. Sloan

**Affiliations:** 1 Drug Discovery Biology Theme, Monash Institute of Pharmaceutical Sciences, Monash University, Parkville, VIC 3052 Australia; 2 Department of Cell & Developmental Biology and Lineberger Comprehensive Cancer Center, School of Medicine, The University of North Carolina Chapel Hill, Chapel Hill, NC 27599 USA; 3 Department of Biomedical Engineering, Duke University, Durham, NC 27708 USA; 4 Department of Mathematics and Statistics, The University of Melbourne, Parkville, VIC 3010 Australia; 5 Cousins Center for PNI, UCLA Semel Institute, and Jonsson Comprehensive Cancer Center, University of California Los Angeles, Los Angeles, CA 90095 USA; 6 Division of Cancer Surgery, Peter MacCallum Cancer Centre, East Melbourne, VIC 3002 Australia; 7 Current address: Peter MacCallum Cancer Centre, East Melbourne, VIC 3002 Australia

## Abstract

**Introduction:**

For efficient metastatic dissemination, tumor cells form invadopodia to degrade and move through three-dimensional extracellular matrix. However, little is known about the conditions that favor invadopodia formation. Here, we investigated the effect of β-adrenoceptor signaling - which allows cells to respond to stress neurotransmitters - on the formation of invadopodia and examined the effect on tumor cell invasion.

**Methods:**

To characterize the molecular and cellular mechanisms of β-adrenergic signaling on the invasive properties of breast cancer cells, we used functional cellular assays to quantify invadopodia formation and to evaluate cell invasion in two-dimensional and three-dimensional environments. The functional significance of β-adrenergic regulation of invadopodia was investigated in an orthotopic mouse model of spontaneous breast cancer metastasis.

**Results:**

β-adrenoceptor activation increased the frequency of invadopodia-positive tumor cells and the number of invadopodia per cell. The effects were selectively mediated by the β_2_-adrenoceptor subtype, which signaled through the canonical Src pathway to regulate invadopodia formation. Increased invadopodia occurred at the expense of focal adhesion formation, resulting in a switch to increased tumor cell invasion through three-dimensional extracellular matrix. β_2_-adrenoceptor signaling increased invasion of tumor cells from explanted primary tumors through surrounding extracellular matrix, suggesting a possible mechanism for the observed increased spontaneous tumor cell dissemination *in vivo*. Selective antagonism of β_2_-adrenoceptors blocked invadopodia formation, suggesting a pharmacological strategy to prevent tumor cell dissemination.

**Conclusion:**

These findings provide insight into conditions that control tumor cell invasion by identifying signaling through β_2_-adrenoceptors as a regulator of invadopodia formation. These findings suggest novel pharmacological strategies for intervention, by using β-blockers to target β_2_-adrenoceptors to limit tumor cell dissemination and metastasis.

## Introduction

Metastasis is the main cause of death from cancer, and involves the dissemination of cancer cells from the primary tumor to colonize distant tissues [1]. To aid dissemination, cancer cells form specialized actin-rich structures called invadopodia that facilitate invasion through the basement membrane and surrounding stroma [2]. Invadopodia produce and localize matrix metalloproteases (MMPs) to focally degrade surrounding extracellular matrix. Src has been shown to play a key role in invadopodia formation and invasion [3, 4]. Src may be recruited away from focal adhesions [5, 6], resulting in a shift to invadopodia-mediated invasion, which favors tumor cell movement through the surrounding three-dimensional extracellular matrix and leads to tumor cell dissemination to distant organs.

Tumor progression and metastasis are regulated by bidirectional signaling between tumor cells and their surrounding microenvironment [7]. β-adrenoceptors (βARs) are found on both tumor cells and untransformed cells in the tumor microenvironment, and allow for cellular response to neural signals [8, 9]. Neurotransmitters including noradrenaline and adrenaline are released during stress and bind to βARs [10]. Activation of βAR induces intracellular signaling cascades that accumulate cAMP, activate PKA, and regulate gene transcription to modify cell behavior [11]. βAR signaling drives metastasis [12–14], and Src activation has been implicated in βAR regulation of metastasis [15]. However, the cellular mechanisms by which βARs drive tumor cell dissemination remain elusive.

To investigate, we explored the effect of βAR signaling on the molecular and cellular mechanisms of tumor cell invasion. Using functional assays we found that βAR signaling enhanced invadopodia formation through canonical Src signaling pathways. Increased invadopodia formation was associated with loss of focal adhesions and enhanced tumor cell invasion through three-dimensional extracellular matrix. These effects were selectively mediated by the β_2_AR subtype, which enhanced tumor cell invasion from primary mammary tumors and increased metastasis *in vivo*. β_2_AR regulation of invadopodia could be reversed by pharmacological blockade, suggesting a strategy to reduce tumor cell dissemination in breast cancer.

## Methods

### Reagents

CGP-20712A dihydrochloride, ICI-118551 hydrochloride, and xamoterol hemifumerate were sourced from Tocris Bioscience (Bristol, UK); formoterol fumarate from BioNet (Cornwall, UK); and PP2 Src inhibitor from Calbiochem (Alexandria, Australia). Agonists were used at 0.5 μM and antagonists at 0.05 μM unless otherwise stated. Other reagents and chemicals were from Sigma-Aldrich (Castle Hill, Australia) unless otherwise stated.

### Cell culture and transduction

The highly metastatic HM variant of MDA-MB-231 breast cancer cell line (described throughout as MDA-MB-231) was a kind gift from Dr Zhou Ou, Fudan University Shanghai Cancer Center, China [16, 17]. The cell line identity was verified by karyotyping (CellBank Australia, Westmead, NSW Australia) and transduced to express codon-optimized luciferase 2, *luc2* [13]. Cells were cultured in Dulbecco’s modified Eagle’s medium (DMEM; Invitrogen) supplemented with 10 % fetal bovine serum (FBS). Cells were maintained at 37 °C, in a humidified environment with 5 % CO_2_. The 66cl4 mouse mammary adenocarcinoma cell line (a kind gift from Prof Robin Anderson, Peter MacCallum Cancer Centre, East Melbourne, VIC, Australia) was cultured in α-minimum essential medium (Invitrogen, Scoresby, VIC) containing 10 % FBS [12]. These cell lines are characteristic of triple-negative breast cancer [18]. pLL5.0-LifeAct-GFP-2A-luc2 was generated by ligation of a sequence encoding the 2A cleavage peptide and *luc2* immediately 3′ of green fluorescent protein (GFP) in pLL5.0-LifeAct-GFP [19]. The *luc2* sequence was PCR amplified from pGL4.10 (Promega, Madison, WI USA) using a modified 5′ primer that encoded the 2A peptide sequence [20]. The product was sequence validated. Lentiviral production was performed as described previously [21] and a fluorescent population identified by fluorescence-activated cell sorting.

### Gene expression

RNA was extracted using the RNeasy kit (Qiagen Chadstone, VIC Australia) and gene expression was quantified by quantitative RT-PCR using the iScript One-Step RT-PCR kit (Bio-Rad, Gladesville, NSW Australia) and Taqman probes (*ADRB1*, Hs02330048_s1; *Adrb1*, Mm00431701_s1; *ADRB2*, Hs00240532_s1; *Adrb2*, Mm02524224_s1; *ADRB3*, Hs00609046_m1; *Adrb3*, Mm02601819_g1; Life Technologies, Tullamarine, VIC Australia) and run on a CFX96 Real Time System (Bio-Rad). Triplicate determinations were evaluated by threshold cycle analysis and expressed relative to the housekeeping gene (*ACTB*, Hs99999903_m1; *Actb*, Mm00607939).

### cAMP assay

cAMP accumulation was quantified by the Alphascreen cAMP kit (Perkin Elmer, Melbourne, VIC Australia) as per the manufacturer’s instructions. Cells were serum-starved overnight and treated with antagonists in stimulation buffer for 30 minutes before addition of agonists for 10 minutes at 37 °C. Cells were lysed in ice-cold ethanol, which was evaporated before reconstitution in detection buffer for cAMP assay.

### Fixed invadopodia assay

MDA-MB-231 cells expressing LifeAct-GFP-Luc2 were serum-starved overnight. Cells were preincubated with antagonists for 20 minutes before plating onto coverslips coated with Alexa Fluor-tagged gelatin or fibronectin in media containing 10 % serum, ± agonists or antagonists for 5 hours. Cells were fixed in 4 % paraformaldehyde and nuclei counterstained with 1 μg/ml Hoechst 33242. Cells were imaged on an SP8 confocal microscope (Leica Microsystems, North Ryde, NSW Australia) using a 63× PL APO CS2 1.4NA objective with excitation at 405 nm, 488 nm, 561 nm, and 633 nm and emission detectors set as follows: 415-485 PMT, 495-535 HyD, 570-635 PMT, and 645-705 PMT. Images were captured using LAS AF software version 3.2 (Leica Microsystems). Invadopodia were defined as actin-positive puncta overlying degraded (fluorescence-negative) matrix. The frequency of invadopodia-positive cells was manually counted in eight random fields of view from three independent experiments. *N* ≥80 cells per treatment were quantified for each experiment. To determine the number of invadopodia per cell, image stacks were prepared and submitted to the Invadopodia Analysis Server [19], which uses a high-pass filter and threshold to identify regions of high actin concentration that colocalize with matrix degradation to identify active invadopodia. Average matrix fluorescence outside the cell bodies was set to 500 arbitrary units to allow the local difference values to be compared between extracellular matrix preparations [19]. Images were not preprocessed prior to submission to the Server. *N* ≥130 cells per treatment were quantified for each experiment. Experiments were conducted in triplicate.

### Immunofluorescence

Focal adhesions were localized in MDA-MB-231 cells following fixation and permeabilization by incubating with 10 μg/ml anti-paxillin antibody (clone 5H11; Millipore, Bayswater, VIC Australia), followed by 1 μg/ml Alexa Fluor 488-conjugated secondary antibody and 200 U/ml Alexa Fluor-647 phalloidin (Invitrogen) to co-stain actin. Nuclei were counterstained with 1 μg/ml Hoechst 33242. Cells were imaged using an SP8 confocal microscope as already described. Focal adhesions were measured in ImageJ software (National Institutes of Health, Bethesda, MD USA) by drawing a region of interest across the longest axis of each paxillin-positive focal adhesion at the cell perimeter. Only adhesions greater than 3 pixels long were counted. Focal adhesions were analyzed from four random fields of view from each of two independent experiments. No nonlinear adjustments were made during image processing and all adjustments were applied to the entire image. Images were cropped to a single cell for presentation in figures without removing any additional image information. β_2_AR was localized by immunostaining as described previously [12] in de-identified human breast cancer tissue obtained under approval from the Institutional Review Board. Samples were counterstained with anti-macrophage antibody (0.7 μg/ml Ham56; Dako, North Sydney, NSW Australia) to distinguish tumor cells from stromal cells.

### Two-dimensional migration assay

Serum-starved LifeAct-GFP^+^ MDA-MB-231 cells were seeded on fibronectin-coated chamber slides and treated ± agonists/antagonists. Cells were imaged on a Ti-Ex microscope (Nikon Instruments, Melville, NY USA) using a 20× Plan Apo 0.6NA objective with fluorescence filters GFP excitation 470/40 and emission 525/50, red fluorescence excitation 545/30 and emission 620/60, and far red excitation 620/60 and emission 700/75. The microscope was fitted with an incubation chamber heated to 37 °C and humidified CO_2_ was supplied to the cells. Images were captured every 20 minutes for 16 hours using a SPOT Pursuit camera (Diagnostic Instruments, Victoria Park, WA Australia) and MetaMorph 7.8.0 software (Molecular Devices, Sunnyvale, CA USA). Time-series images were reconstructed and cells tracked manually using ImageJ software. No nonlinear adjustments were made during image processing and all adjustments were applied to the entire image. The experiment was conducted in duplicate.

### Single-cell three-dimensional migration assay

Serum-starved LifeAct-GFP^+^ MDA-MB-231 cells were resuspended in 1 mg/ml collagen (type I rat tail; Merk-Millipore) ± agonists/antagonists and the matrix was set at 37 °C for 20 minutes. Cells were imaged every 20 minutes for 16 hours using an A1R confocal microscope (Nikon) using a 20× Plan Apo 0.6NA objective with a correction collar, with excitation 488 nm and an emission 505–545 BP filter. The microscope was fitted with an incubation chamber heated to 37 °C and humidified CO_2_ was supplied to cells. Z-stacks of 100 μm in 20 μm steps were captured using NIS Elements software (version 3.22; Nikon Instruments). Three-dimensional images were reconstructed in Imaris 7.6.4 software (Bitplane, Zurich Switzerland) and cell migration was tracked. Protrusion-positive cells were counted manually 6 hours after seeding. Any cell with a protrusion longer than half the cell body diameter was considered protrusion-positive. Images were prepared as maximum intensity projections. No nonlinear adjustments were made during image processing and all adjustments were applied to the entire image. Images were cropped to a single cell for presentation in figures without removing any additional image information. *N* ≥20 cells were quantified per treatment. Experiments were conducted in triplicate.

### Metastasis models and explants

All procedures involving mice were carried out under protocols approved by the Institutional Animal Ethics Committee and in accordance with National Health and Medical Research Council animal ethics guidelines. MDA-MB-231 breast cancer cells were injected into the left fourth mammary fat pad of BALB/c nude mice as described previously [12]. Formoterol (5 mg/kg/day) or saline placebo was injected once daily by subcutaneous injection. Bioluminescence imaging was used to track tumor growth and metastatic progression using an IVIS Lumina II (Perkin Elmer) as described previously [12, 17]. Metastasis was confirmed by *ex vivo *imaging and hematoxylin and eosin staining. For analysis of tumor cell invasion from explanted primary tumors, a subset of mice were anesthetized on day 9 of mammary tumor growth and euthanized. Tumors were surgically removed from the mammary fat pad and embedded in 1 mg/ml collagen ± 0.5 μM formoterol. Explants were maintained at 37 °C with 5 % CO_2_ and imaged every 2 days over a period of 8 days using an A1R confocal microscope (Nikon) as already described. Z-stacks were captured over 250 μm at 50 μm steps with large image format stitching of a 7 × 7 field of view grid. Time series images were reconstructed and analyzed using the Fiji distribution of ImageJ with StackReg plugin. Tumor boundaries were defined on the image that was acquired on day 0 and the area of tumor cell invasion of surrounding extracellular matrix outside this region of interest was then determined at subsequent time points. Five tumors were quantified per treatment. Two experimental replicates were conducted.

### Statistical analyses

Generalized linear models were used to assess the relationship between treatments and experimental response [22]. The number of invadopodia per cell and the frequency of invadopodia-positive cells were modeled by binomial (logit link) and Poisson (log link) generalized linear models, respectively. Treatment differences for single continuous responses such as cell displacement and cAMP accumulation were analyzed by analysis of variance. For statistical analysis of *in vivo* metastatic progression and *ex vivo* invasion, the impact of β_2_AR stimulation over time was analyzed using the linear model:

$$ \ln (Y)=\upbeta 0 + \upbeta 1t + \upbeta 2I\left\{\mathrm{formoterol}\right\}x\ t + \varepsilon $$

where *Y* is the luciferase activity (total flux, Fig. 5b) or the area of tumor cell invasion outside the tumor boundaries (Fig. 5c), β0 is the intercept parameter, *t* is time, *I*{formoterol} (taking values 0 or 1) indicates the presence of formoterol, and ε is a Gaussian random error. For the fitted models, we report estimated regression coefficients quantifying the relationship between the response and treatments, and the corresponding *p* values for testing the null hypothesis of no treatment effect. For experiments involving multiple treatments, we tested simultaneous differences between pairs of treatment effects (Tukey’s all-pair comparisons) [23]. Confidence intervals and *p* values were adjusted at the nominal 5 % significance level for multiple testing. Statistical analysis was carried out in the R computing environment [24]. Generalized linear models were fitted using the routine glm while multiple comparisons used the routine glht in the package multicomp [25].

## Results and Discussion

**Fig. 1:**
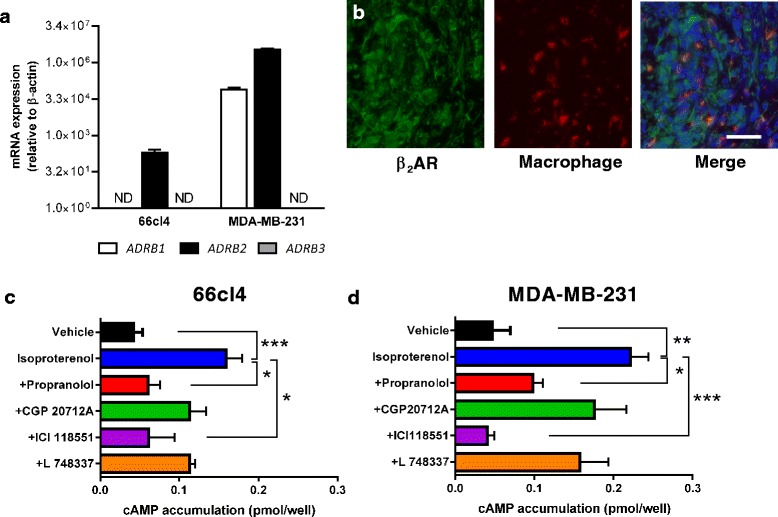
Breast cancer cells have functional β_2_-adrenoceptors (*β*_*2*_ *AR*). **a** *ADRB1*, *ADRB2*, and *ADRB3* mRNA transcript levels were quantified by quantitative RT-PCR, with expression normalized to *ACTB*. **b** Immunostaining of β_2_AR and macrophages in archival breast cancer tissue. Scale bar: 100 μm. **c**, **d** cAMP accumulation was quantified in **c** 66cl4 cells and **d** MDA-MB-231 cells after treatment with vehicle, or 10 nM isoproterenol ± 1 μM antagonists including propranolol (nonselective), CGP-20712A (β_1_AR selective), ICI-118551 (β_2_AR selective), or L748337 (β_3_AR selective). *N* = 3. Error bars: standard error of the mean (SEM). ND: not detected. *p <0.05 and **p <0.01

We first confirmed expression of βAR in MDA-MB-231 and 66cl4 tumor cells, which are breast cancer cells lines that respond to stress signaling with increased metastasis from primary orthotopic mammary tumors [12, 13]. Expression analyses found higher β_2_AR than β_1_AR transcription in MDA-MB-231 cells, while 66cl4 expressed only β_2_AR (Fig. 1a). β_3_AR was not detectable in either cell line, although priming from these probes has been confirmed [26, 27]. Immunostaining confirmed that tumor cells from an archival clinical sample expressed β_2_AR, suggesting that findings may be relevant to human breast cancer (Fig. 1b). To evaluate receptor functionality, we treated tumor cells with βAR ligands and quantified the effect on intracellular cAMP accumulation. In each cell line, treatment with the nonselective βAR agonist isoproterenol increased cAMP accumulation (Fig. 1c, d), consistent with previous studies [28]. This effect was blocked by the nonselective βAR antagonist propranolol or the β_2_AR-selective antagonist ICI-188551. Treatment with β_1_AR-selective antagonist CGP-20712A or β_3_AR-selective antagonist L748337 did not block cAMP accumulation (Fig. 1c, d). These findings confirm that β_2_AR is the dominant functional βAR in these breast cancer cell lines.

**Fig. 2:**
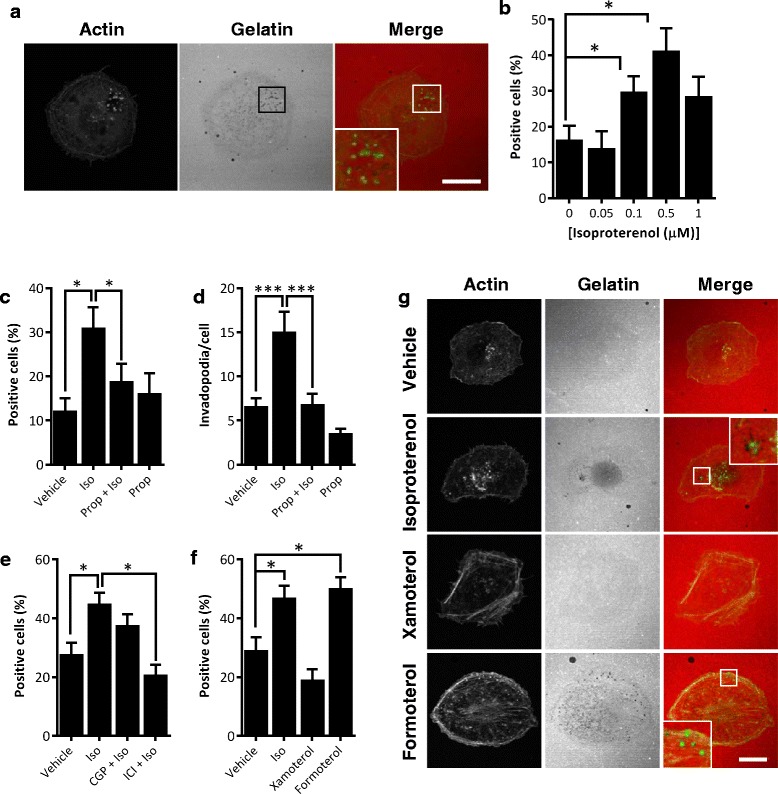
β_2_AR signaling induces invadopodia formation. **a** LifeAct-GFP^+^ MDA-MB-231 cells were plated on Alexa-568-labelled gelatin and active invadopodia were identified by confocal microscopy as LifeAct-GFP^+^ puncta colocalized with degraded gelatin (loss of red fluorescence; see inset). Representative images are shown. Scale bar: 20 μm, or 5 μm for inset panel. **b** The frequency of invadopodia-positive cells was determined in cells treated with isoproterenol (*Iso*). **c**, **d** Cells were treated with 0.5 μM *Iso* ± 0.05 μM propranolol (*Prop*), and **c** the frequency of invadopodia-positive cells was determined or **d** the number of invadopodia per cell was determined (*N* >130 cells per treatment). **e** Cells were treated with Iso ± β_1_AR-selective antagonist CGP-20712A (*CGP*) or β_2_AR-selective antagonist ICI-118551 (*ICI*) and the effect on invadopodia formation was quantified. **f**, **g** Cells were treated with Iso, β_1_AR-selective agonist xamoterol, or β_2_AR-selective agonist formoterol and **f** the effect on invadopodia was quantified. **g** Representative confocal sections are shown. Inset shows GFP+ active invadopodia on degraded matrix (loss of red fluorescence). Scale bar: 20 μm, or 5 μm for inset panels. *N* > 80 cells per treatment unless otherwise stated. Experiments were conducted in triplicate. Error bars: SEM. *p <0.05, **p <0.01 and ***p <0.001

To investigate the role of βAR signaling in invadopodia formation, LifeAct-GFP^+^ MDA-MB-231 cells were plated on a fluorescent gelatin matrix and the effect of β-agonist isoproterenol on matrix degradation was evaluated (Fig. 2a). While actin-rich puncta were identified in the entire population, only 16 % (± 4 %) of tumor cells produced active invadopodia as defined by degradation of underlying matrix fluorescence (Fig. 2a, b). Treatment with isoproterenol led to an increase in the frequency of invadopodia-positive cells, with a maximum increase of 2.5-fold in response to 0.5 μM isoproterenol (Fig. 2b). In addition to increasing the frequency of invadopodia-positive cells in the population (Fig. 2b, c), isoproterenol also increased the average number of invadopodia per cell by 2.7-fold (Fig. 2d). Treatment with the nonselective β-blocker propranolol blocked the effect of isoproterenol on both the frequency of invadopodia-positive cells and the number of invadopodia per cell (Fig. 2c, d). This confirms that isoproterenol signals via βAR to increase invadopodia formation. To confirm the βAR subtype that mediates this effect, MDA-MB-231 cells were treated with selective antagonists and the effect on invadopodia was evaluated. β_2_AR-selective antagonist ICI-118551 blocked the increase in invadopodia formation in response to isoproterenol, whereas there was no effect of β_1_AR-selective antagonist CGP-20712A (Fig. 2e). To determine whether β_2_AR signaling is sufficient to induce invadopodia formation, cells were treated with selective βAR agonists. β_2_AR-selective agonist formoterol – but not β_1_AR-selective agonist xamoterol – induced invadopodia formation (Fig. 2f, g), confirming a key role for β_2_AR signaling in the formation of invadopodia in these cells.

**Fig. 3:**
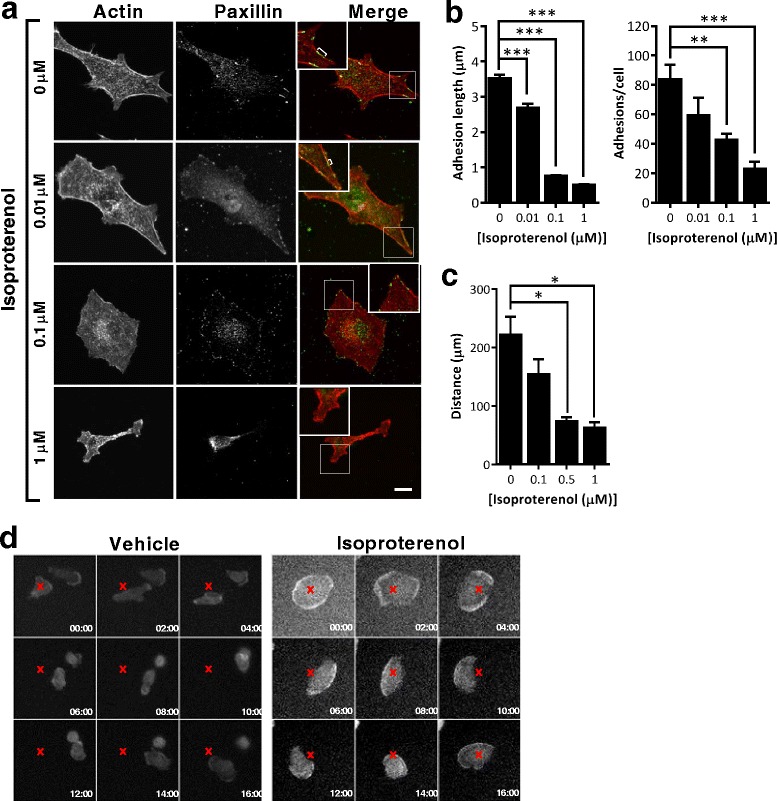
β_2_AR signaling decreases focal adhesions and decreases migration on two-dimensional surfaces. **a** Representative confocal sections of tumor cells stained for actin and paxillin to identify focal adhesions. Scale bar: 10 μm. *Square brackets* in inset identify focal adhesions. **b** The effect of isoproterenol on focal adhesion length and number of adhesions per cell was quantified. **c** The effect of isoproterenol on cell migration on two-dimensional fibronectin surfaces over time was quantified. **d** Representative time series images of cells treated with vehicle or isoproterenol. *Red cross* marks the location of the cell at the commencement of treatment. Time in hours is indicated. *N* > 20 cells per condition. Error bars: SEM. *p <0.05, **p <0.01 and ***p <0.001

Formation of invadopodia requires adhesion proteins that may be sequestered from focal adhesions [6]. To investigate the effect of βAR signaling on focal adhesions we treated breast cancer cells with isoproterenol and used immunostaining to quantify the number and length of paxillin-positive focal adhesions (Fig. 3a). Isoproterenol resulted in a concentration-dependent decrease in adhesion length and decreased number of focal adhesions per cell (Fig. 3a, b). Loss of focal adhesion-associated proteins has been linked to a decreased frequency of focal adhesions and reduced capacity for cell migration on two-dimensional surfaces [29]. Consistent with the change in focal adhesions having a functional effect on cell movement, isoproterenol decreased the migration of cancer cells on a two-dimensional fibronectin surface (Fig. 3c, d).

**Fig. 4:**
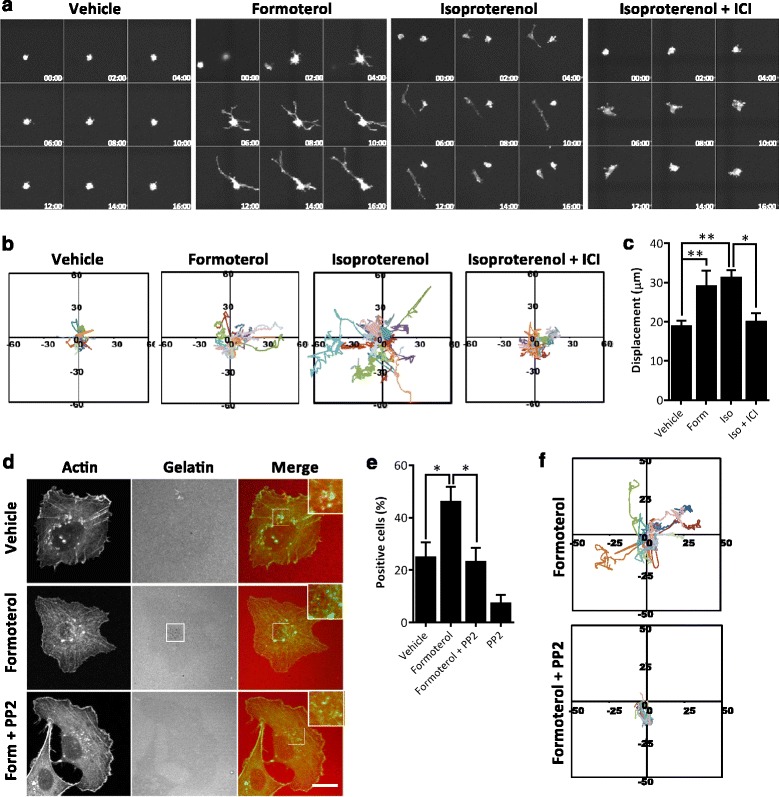
β_2_AR-induced invadopodia formation is dependent on Src and increases tumor cell invasion in a three-dimensional collagen matrix. **a** Representative time series images acquired by confocal microscopy showing the effect of formotorol or isoproterenol (*Iso*) ± 0.05 μM ICI-118551 (*ICI*) on development of cell protrusions. Time in hours is indicated. **b** Graphical representation of track measurements is shown for 20 cells in each condition. **c** Quantification of cell displacement from the point of origin. **d**, **e** Representative maximum intensity projections of LifeAct-GFP^+^ MDA-MB-231 cells treated with 0.5 μM PP2 ± 0.5 μM formoterol and examined for active invadopodia. Inset shows GFP+ green puncta (active invadopodia that have degraded underlying matrix) or yellow puncta (inactive invadopodia where GFP+ puncta have not degraded the underlying red fluorescent matrix). Scale bar: 10 μm, or 5 μm for inset panels. **e** The percentage of invadopodia positive cells was quantified. *N* >80 cells per condition. **f** Graphical representation of track measurements after cells were embedded in 1 mg/ml collagen matrix and treated with 0.5μΜ formoterol ± PP2 Src inhibitor. *N* = 20 cells per condition. Error bars: SEM. *p <0.05 and **p <0.01

These findings suggest that β_2_AR signaling induces an invasive phenotype that favors tumor cell invasion in three-dimensional environments. To investigate this, MDA-MB-231 breast cancer cells were embedded in collagen matrix and cell locomotion was tracked over time. Under control conditions, cells remained rounded with little displacement from the position of origin (Fig. 4a, b). Treatment with β_2_AR-selective agonist formoterol induced cancer cells to elongate and form protrusions (Fig. 4a) and increased cell displacement over time (Fig. 4b, c), indicating that β_2_AR signaling is sufficient to induce invasion of MDA-MB-231 breast cancer cells. Treatment with isoproterenol similarly induced cellular protrusion formation and these effects were blocked by the β_2_AR-selective antagonist ICI-118551, confirming a role for β_2_AR signaling in these effects on cell invasion through a three-dimensional collagen matrix. By identifying differential effects of βAR signaling between cell movement on two-dimensional surfaces compared with migration in three-dimensional environments, these findings may reconcile seemingly inconsistent observations for the effects of endogenous neurotransmitters and βAR-selective agonists on tumor cell migration [30–33]. Because movement in three-dimensional environments reflects processes that are required for tumor cell intravasation and extravasation during metastasis, these findings suggest a cellular mechanism for observations that βAR signaling drives breast cancer progression [12, 13].

Src activity plays a key role in invadopodia formation [4, 6], and has been implicated in βAR regulation of ovarian cancer invasion, although the cellular mechanisms are unclear [15]. To investigate the role of Src in β_2_AR regulation of invadopodia, MDA-MB-231 cells were pretreated with the Src inhibitor PP2 prior to treatment with formoterol. PP2 treatment blocked the effect of β_2_AR stimulation on the frequency of invadopodia (Fig. 4d, e), as seen by loss of matrix fluorescence. Treatment with PP2 alone did not significantly change the frequency of invadopodia compared to treatment with vehicle. Treatment with PP2 also blocked the effect of formoterol on invasion of cells in three-dimensional collagen matrix (Fig. 4f), demonstrating that Src is essential for β_2_AR-mediated invasion. These findings indicate that β_2_AR signaling regulates invadopodia formation and tumor cell invasion through canonical Src signaling pathways. βAR activation was recently shown to phosphorylate Src at Y^416^ in addition to S^17^ [15], suggesting that characterization of the activation status of Src will contribute to a more complete understanding of the molecular mechanisms involved. Additional insight may be gained from clarifying other β_2_AR-induced molecular changes that may amplify effects on tumor cell dissemination. For example, norepinephrine was shown to protect cancer cells from anoikis by activating and relocalizing focal adhesion kinase [34]. Coordinated regulation of invadopodia formation and reduced anoikis in response to β_2_AR-mediated neural signaling may serve to amplify the effects on tumor cell dissemination and cancer progression.

**Fig. 5:**
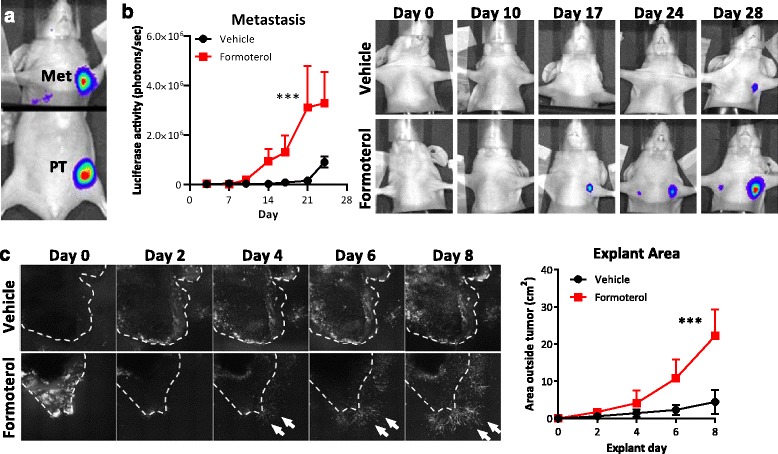
β_2_AR signaling induces tumor cell invasion and metastasis from primary mammary tumors. **a** Representative image of the orthotopic metastasis model. Luciferase-tagged tumor cells were injected into the fourth mammary fat pad (*PT*) and spontaneous metastases to the lymph node and lung (*Met*) were detected by optical bioluminescence imaging. Lower body exposure: 1 second. Upper body exposure: 60 seconds. *Black bar* separates images taken with two different exposures. **b** Mice were treated daily with 5 mg/kg formoterol (or saline vehicle) during tumor development and the effect of distant metastasis was quantified over time by bioluminescence imaging and expressed relative to primary tumor size. *N* = 5 at each time-point. **c** Primary tumors were resected from the mammary fat pad of vehicle vs. formoterol-treated mice at day 10 after tumor cell injection and embedded in a three-dimensional collagen matrix. Invasion of LifeAct-GFP^+^ tumor cells beyond explant boundaries into the surrounding collagen (arrows) was imaged over 8 days by confocal microscopy and quantified. *N* = 5 at each time point. Error bars: SEM. ***p <0.001

To investigate the impact of β_2_AR regulation of tumor cell invasion *in vivo*, bioluminescence imaging was used to track the effect of β_2_AR-selective agonist formoterol on metastatic progression in an orthotopic xenograft model of breast cancer. Mice were implanted with luciferase-tagged MDA-MB-231 cells into the left fourth mammary fat pad and spontaneous metastasis was tracked by bioluminescence imaging using 1-second exposure to detect signals from primary tumors and 60-second exposure to detect signals from metastases (Fig. 5a). Treatment with formoterol during tumor development accelerated the formation of metastasis (Fig. 5b). To examine whether increased metastasis was linked to increased tumor cell invasion, a subset of mammary tumors were surgically resected 9 days after tumor cell inoculation, before the onset of metastasis, and embedded in a three-dimensional collagen matrix. In contrast to tumors from vehicle-treated mice, which demonstrated minimal invasion of cells into the surrounding extracellular matrix over the 8-day imaging period, tumors from mice treated with formoterol had rapid invasion of tumor cells into the surrounding matrix (Fig. 5c). This shows that β_2_AR signaling increases dissemination from explanted tumors, suggesting a possible mechanism for increased distant metastasis.

While recent studies of βAR-mediated stress biology on cancer progression have focused on the impact of neural signaling on stromal cells in the tumor microenvironment [12, 14, 35, 36], the findings presented here demonstrate that tumor cells are directly responsive to βAR signaling. By driving a shift away from focal adhesions to invadopodia formation, β_2_AR signaling would favor tumor cells with enhanced capacity for invasion through the three-dimensional extracellular matrix of the tumor microenvironment. Metastasis is a highly inefficient process and requires successful completion of a series of inter-connected steps including dissemination from the primary tumor, survival in circulation, extravasation, and colonization at distant sites [37–39]. Failure at any stage of metastasis will result in failure of the entire process [40]. By increasing both the density of invadopodia per cell and the frequency of invadopodia-positive cells in the tumor cell population, β_2_AR signaling may promote metastasis by enhancing the invasive capacity of cells in the tumor.

These findings suggest that blocking tumor cell responsiveness to stress signaling may protect against tumor cell invasion and metastasis. Specifically, the effects of β_2_AR signaling on invadopodia formation were reversed by β-blockade, providing a cellular mechanism for clinical observations that pharmacological β-blockade was linked to improved cancer outcomes [41–43]. Retrospective epidemiological studies found that β-blocker treatment of comorbid hypertension was associated with reduced metastasis and improved survival in different cancer types including breast and prostate cancers and melanoma (reviewed in [44]). The current findings show that β-blockers act directly on cancer cells to decrease their invasiveness. While not excluding that β-blockade regulates cancer progression through effects on the tumor microenvironment (e.g., modulating recruitment of inflammatory cells or angiogenesis) [12, 35], these findings suggest that β-blockers may be optimally targeted to patients with high tumor cell expression of β_2_AR. Recent clinical practice has preferred the use of β_1_-selective blockers for treatment of hypertension, to avoid adverse effects on bronchoconstriction [45]. The findings presented here suggest that use of nonselective β-blockers such as propranolol may be required to favorably impact cancer outcomes. Additionally these findings may warrant the development of novel β_2_-selective blockers that may be targeted to tumor cells to avoid adverse side effects. Finally, these findings suggest that it may be important to consider the effect of clinically used β_2_AR agonists (e.g., by asthmatics for bronchodilation) on cancer progression.

## Conclusions

Here, we present evidence that β_2_AR signaling drives a switch from focal adhesions to invadopodia formation in breast cancer cells to increase cell invasion in three-dimensional environments. These findings suggest that β_2_AR may be a key receptor for transmission of neural signals from the tumor microenvironment to regulate behavior of tumor cells. The findings provide a plausible mechanism for accumulating evidence that chronic stress promotes cancer progression and metastasis [12, 35, 46, 47], and suggest that selective pharmacological blockade of β_2_AR signaling pathways may be important to slow cancer progression.

## Abbreviations

βAR: β-adrenoceptor
DMEM: Dulbecco’s modified Eagle’s medium
FBS: Fetal bovine serum
GFP: Green fluorescent protein
Iso: Isoproterenol
MMP: Matrix metalloprotease
SEM: Standard error of the mean
